# Implants Versus Lipograft: Analysis of Long-Term Results Following Congenital Breast Asymmetry Correction

**DOI:** 10.1007/s00266-022-02843-5

**Published:** 2022-03-16

**Authors:** Vanessa Brébant, Maximilian Weiherer, Vivien Noisser, Stephan Seitz, Lukas Prantl, Andreas Eigenberger

**Affiliations:** 1 University Center of Plastic, Aesthetic, Hand and Reconstructive Surgery, University Hospital Regensburg, Franz-Josef-Strauß-Allee 11, 93053 Regensburg, Germany; 2 Regensburg Medical Image Computing (ReMIC), Ostbayerische Technische Hochschule Regensburg (OTH Regensburg), Regensburg, Germany; 3 Department of Obstetrics and Gynecology, Caritas Hospital St. Josef, University of Regensburg, Regensburg, Germany; 4 Regensburg Center of Biomedical Engineering (RCBE), OTH Regensburg and Regensburg University, Regensburg, Germany

**Keywords:** Congenital breast asymmetry, PROM, Autologous fat injections, Fat grafting, Lipograft, Implant augmentation, Breast augmentation, 3D volumetry, Three-dimensional imaging, Breast-Q^TM^

## Abstract

**Aims:**

Congenital breast asymmetry represents a particular challenge to the classic techniques of plastic surgery given the young age of patients at presentation. This study reviews and compares the long-term results of traditional breast augmentation using silicone implants and the more innovative technique of lipografting.

**Methods:**

To achieve this, we not only captured subjective parameters such as satisfaction with outcome and symmetry, but also objective parameters including breast volume and anthropometric measurements. The objective examination was performed manually and by using the Vectra^®^ H2 photogrammetry scanning system.

**Results:**

Differences between patients undergoing either implant augmentation or lipograft were revealed not to be significant with respect to patient satisfaction with surgical outcome (*p* = 0.55) and symmetry (*p* = 0.69). Furthermore, a breast symmetry of 93 % was reported in both groups. Likewise, no statistically significant volume difference between the left and right breasts was observed in both groups (*p* < 0.41). However, lipograft patients needed on average 2.9 procedures to achieve the desired result, compared with 1.3 for implant augmentation. In contrast, patients treated with implant augmentation may require a number of implant changes during their lifetime.

**Conclusion:**

Both methods may be considered for patients presenting with congenital breast asymmetry.

**Level of Evidence III:**

This journal requires that authors assign a level of evidence to each article. For a full description of these Evidence-Based Medicine ratings, please refer to the Table of Contents or the online Instructions to Authors www.springer.com/00266.

## Introduction

Severe asymmetry is one of the most common reasons to perform breast augmentation in patients under the age of 18 [1]. Congenital breast asymmetry particularly affects young women in puberty, and patients often face corrective surgery at a young age. The long-term outcome of the surgical treatment is therefore crucial.

Implant augmentation is the standard procedure in the treatment of asymmetry, volume deficits, or micromastia [2]. For patients with congenital breast asymmetry, implant augmentation is usually associated with the need to replace the implant several times during the course of their lives. Hence, surgical procedures are required that provide better long-term stability. Over the last few years, some promising techniques have been developed based on autologous fat transfers, with lipograft being one such approach. In previous work, we demonstrated that enriched autologous fat grafting offers good long-term results in patients presenting midface deficiency, with the procedure clearly improving facial volume loss and skin quality. [3]

In this study, we examine the long-term outcomes for patients with congenital breast asymmetry who underwent either lipograft or silicone implant augmentation. Since both surgical methods are described differently in the literature, we present the protocols we implemented below. Regarding the long-term results, we analyze both patient satisfaction and objective parameters, such as postoperative volume difference and symmetry. We hypothesize that breast augmentation with lipograft offers at least similar objective and subjective long-term results as breast augmentation with silicone implants.

## Materials and Methods

### Study Design and Patients

A total of 32 patients with corrected breast asymmetry, either through lipograft or silicone implant, were included in our retrospective cohort study. Of these 32 patients, 16 underwent alloplastic and 16 autologous breast augmentation (*n *= 16). On average, patients were examined 7± 3.2 years after surgery. Inclusion criterion was any kind of congenital breast asymmetry performed in our institution between January 2008 and December 2019. Patients who were treated with both lipograft and silicone implants or who missed follow-up examinations were excluded. The data collection period ran from March 2020 to July 2020 and was approved by the Ethics Committee of the University Hospital of Regensburg (**20-1654-101**). Relevant metadata are displayed in Table 1.

**Table 1:** Comparison of the alloplastic (implant) and the autologous (lipograft) groups

	Implant (*n*=16)		Lipograft (*n*=16)	
	Mean (± SD)	Range	Mean (± SD)	Range
Age* [years]	21 (± 4.9)	16–33	20 (± 3.7)	16–29
BMI [kg/m^2^]**	21.9 (± 2.1)	18–26	24.4 (± 3.8)	20–33
Postoperative [years]	7.2 (± 3.6)	2.6–12	6.5 (± 2.8)	0.9–10.2
Cup size***	–	B–E	–	A–E
Number of operations	1.6 (± 0.6)	1–3	2.9 (±1.3)	1–5

*At the time of first breast surgery

**At the time of last breast surgery

***Postoperative

### Lipograft

Lipograft mainly comprises the three steps harvesting, processing, and injection. Following the S2K guidelines, saline (0.9 %) and adrenaline (1: 200,000) were infiltrated into the tissue 15 minutes before harvesting. According to the study by Sommer [4], the cell vitality of the harvested fat graft is equal at every harvesting site. Hence, the harvesting areas were chosen according to the volume of fat available and the personal wishes of the patient. Harvesting was performed using waterjet-assisted liposuction (HumanMed BodyJet, Schwerin, Germany) and harvesting cannulas. The harvested adipose tissue is composed of adipocytes and stromal vascular fraction cells, including adipose-derived stem cells (ASCs), pre-adipocytes, fibroblasts, vascular endothelial cells, and a variety of immune cells [5].

During the processing step, as much blood, serum, and tumescent solution as possible was separated from vital mature adipocytes and adipose progenitor cells. This was performed using sedimentation first (~5 minutes) and then centrifugation (3000 rpm for 2 minutes) in a second step. After discarding the unneeded fractions, the processed fat was transplanted as uniformly as possible through multiple 2 mm stab incisions using LuerLock syringes. On average, 213.8 ml of the lipograft was transplanted per session, each of which lasting an average of 103.8 ± 37.2 minutes. Often, multiple sessions were required per patient (2.9 ± 1.3), resulting in an average of 614.7 ml of transplanted lipograft per patient.

### Implant Augmentation

With over 1.8 million surgeries each year, implant-based breast augmentation is the most common esthetic surgical procedure worldwide. The location of implant placement depends on the individual subcutaneous fat layer in patients and is either subpectoral or subglandular. Seven of our 16 patients undergoing breast augmentation with silicone implants received subglandular implants and the other nine subpectoral. In order to achieve a natural-looking result, the scarring should be hidden in the inframammary fold. The average volume of the implants chosen for augmentation was 235.6 ml (ranging from 150 to 355 ml). Each operation lasted an average of 110 ± 37.8 minutes. We demonstrated in a recent study that textured breast implants cause topographic changes, particularly to the upper quadrants of the breast [6].

### Objective Outcome Measurements

**Fig. 1:**
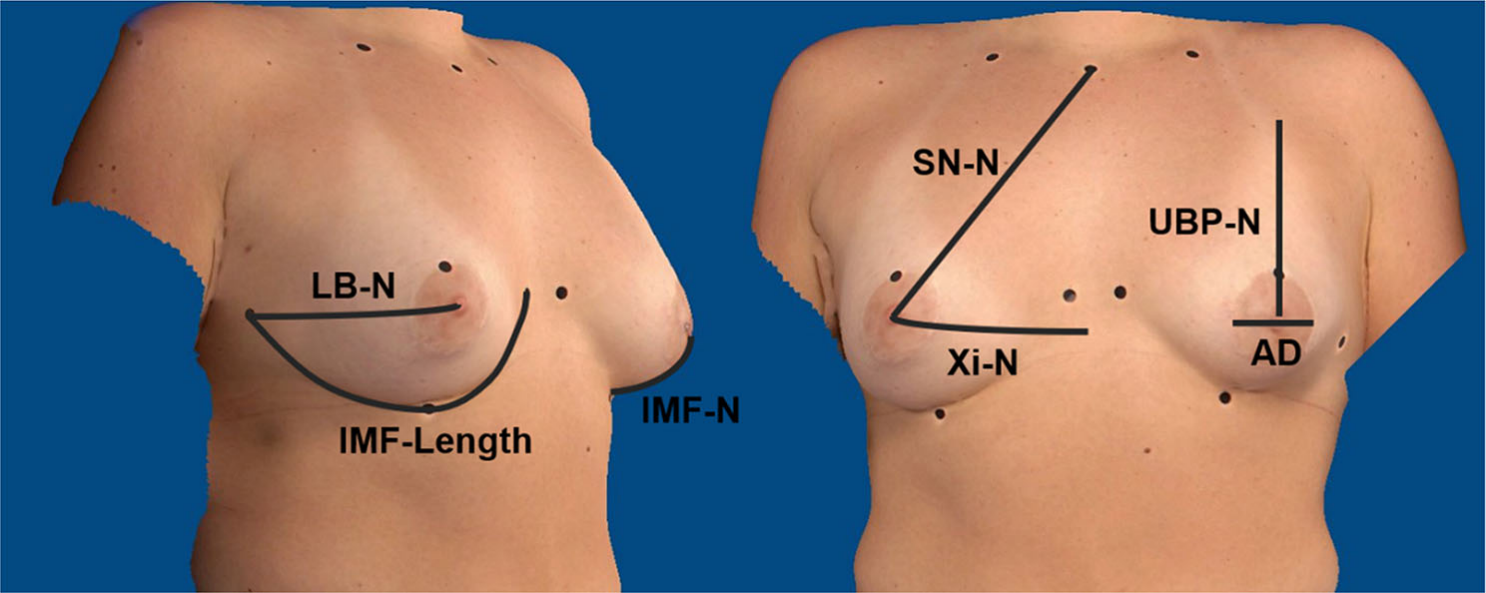
The anthropometric measurements used to calculate SI: LB-N: lateral breast pole to nipple, IMF-Length: inframammary fold length, IMF-N: inframammary fold to nipple, SN-N: sternal notch to nipple, Xi-N: xiphoid to nipple, UBP-N: upper breast pole to nipple, AD: areolar diameter

During the long-term follow-up, the surgical results were evaluated by assessing two objective outcome parameters: symmetry and volume difference between both breasts. Thus, all patients were measured manually using a classic tape measure along the skin surface. The symmetry index (SI) quantified breast symmetry. According to Hartmann et al. [7], the SI calculates the symmetry, returning a value between 0 (worst) and 1 (best) by means of seven anthropomorphic measurements, as illustrated in Fig. 1.

**Fig. 2:**
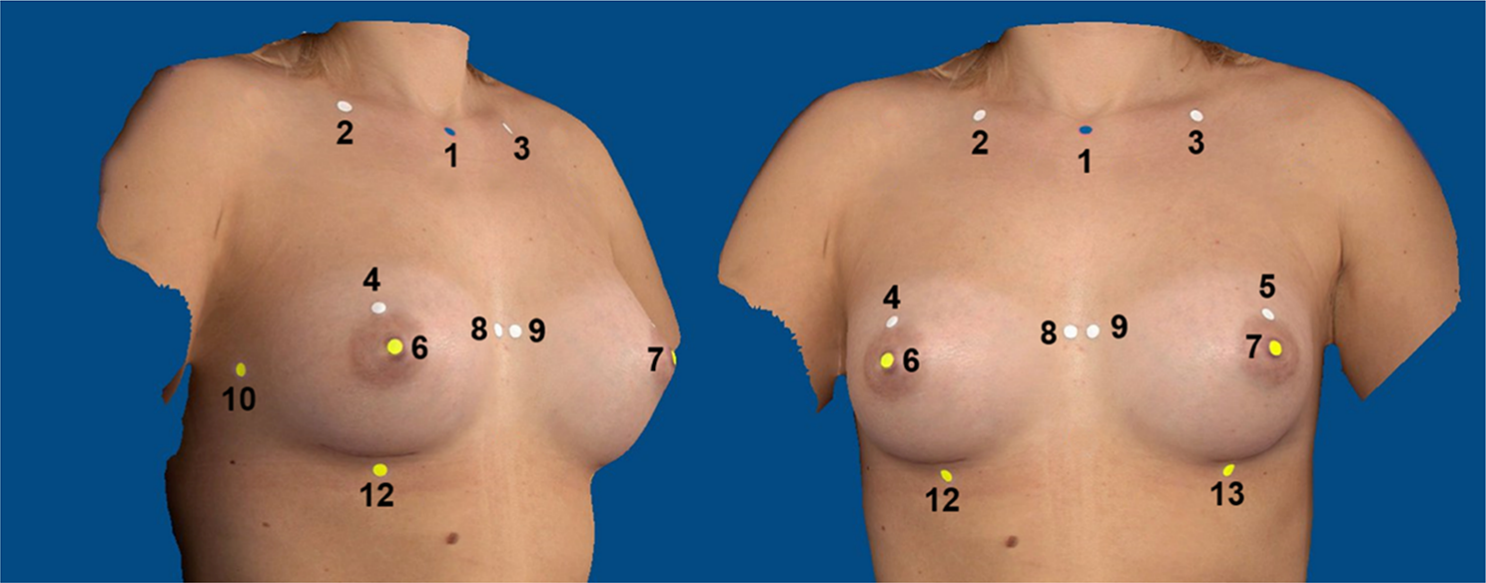
Reference points for Canfield Vectra^®^ H2 (picture created with Canfield Vectra^®^ H2 and edited). 1: sternal notch, 2–3: midpoint of the clavicle, 4–5: most cranial point of the areola, 6–7: nipple, 8–9: end of medial inframammary fold, 10–11: end of lateral inframammary fold, 12–13: most caudal point of the inframammary fold

Post-operative breast volume was calculated using the Breast-V formula as proposed by Longo et al. [8] for ptotic breasts and extended by Huang et al. [9] for non-ptotic breasts. Both formulae have been used in several studies previously [10–13]. In addition, we measured breast volumes and various breast dimensions using the portable Vectra^®^ H2 (Canfield Scientific, USA) photogrammetry scanning system. This system allows us to reproduce the total surface information for an individual patient. The system is supplied with Vectra^®^ Breast-Sculptor^TM^ (Canfield Scientific, USA) software for point cloud processing and mesh generation. This application allows analysis of the 3D model in terms of breast volume and various breast dimensions, as previously demonstrated in a number of studies [14–22]. Thus, 13 points were marked on the patient and subsequently selected in Breast-Sculptor^TM^ (see Fig. 2).

### Patient-Related Outcome Measures

To measure the patient-related outcome, we implemented the Breast-Q™ questionnaire (Breast-Q^TM^ version 2.0^©,^ Augmentation Modules Pre- and Postoperative Scales, The University of British Columbia, licensed for non-profit users by Memorial Sloan Kettering Cancer Center and translated by Mapi Research Trust, 2008). This validated questionnaire is commonly used in similar studies to determine patient satisfaction after breast augmentation [23, 24]. The Breast-Q^TM^ defines the degree of satisfaction as a percentage, with 0 % being the worst and 100 % the best possible value. In addition to the Breast-Q^TM^, patient satisfaction with breast symmetry was polled retrospectively using a scale from 1 (least) to 4 (greatest).

### Statistical Analysis

SPSS^®^ Statistics version 25.0.0. (IBM^®.^, Armonk; New York) was used to perform statistical analysis. Since our data did not follow a normal distribution (Kolmogorov–Smirnov test and the Shapiro–Wilk test) in both groups and in any of the cases, we applied the Mann–Whitney test to detect differences between the groups. *P*-values < 0.05 were considered as statistically significant.

## Results

**Fig. 3:**
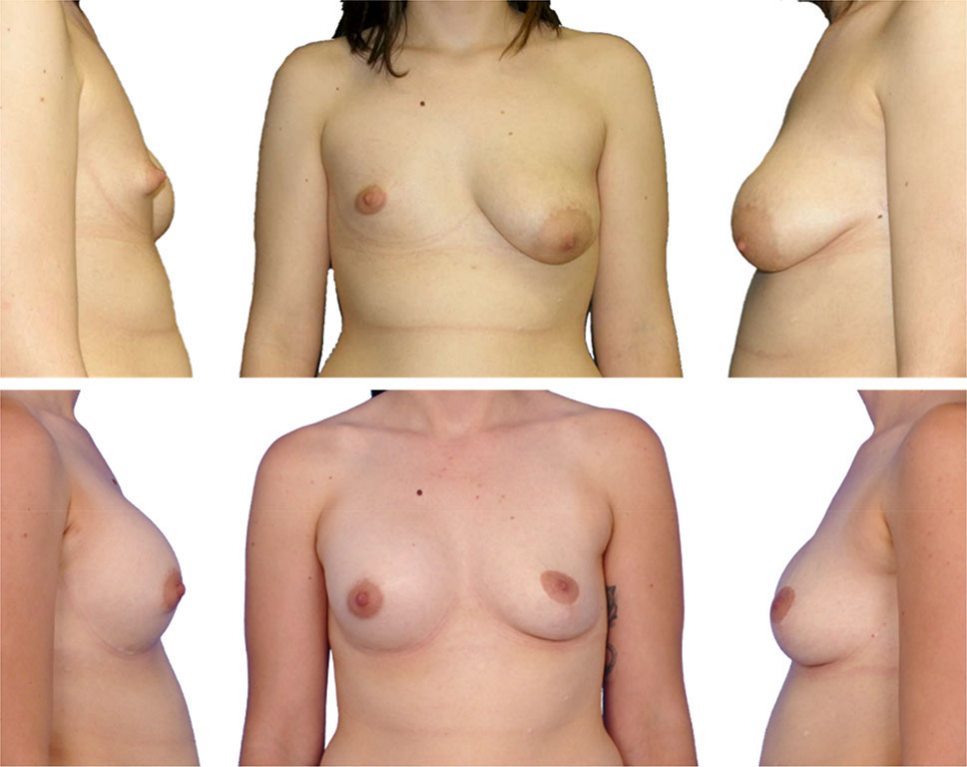
Patient with congenital breast asymmetry and tubular deformity. Top: prior to surgery, 2017. Bottom: postoperative status, 2020, after unilateral breast augmentation with silicone implant and contralateral mastopexy

**Fig. 4:**
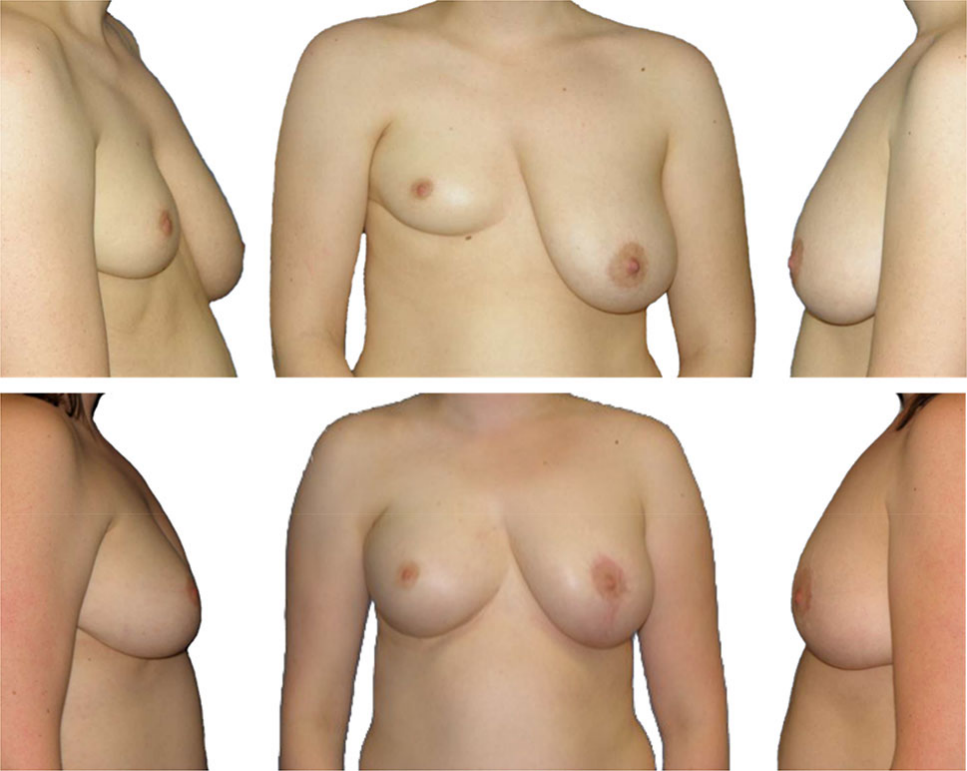
Patient with breast asymmetry and Poland's syndrome. Top: State prior to lipograft therapy in two sessions (surgeries in 2010 and 2013). Bottom: postoperative state, 2020, after additional contralateral mastopexy

Data including the satisfaction with surgical outcome, the SI, and the difference in volume were collected for all 32 patients. As an example, Figs. 3 and 4 illustrate a pre- and postoperative comparison for both surgical methods.

### Patient Satisfaction with the Outcome of Surgical Intervention

**Fig. 5:**
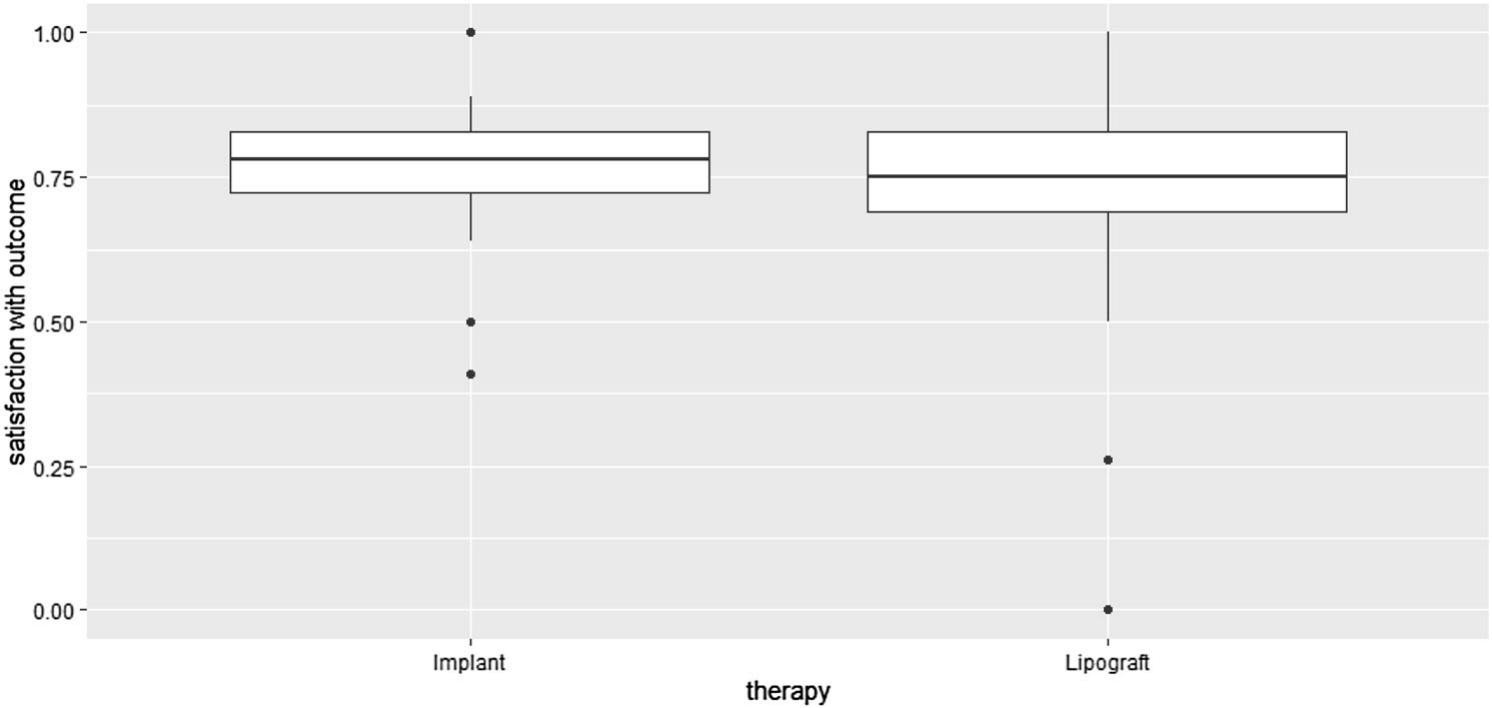
Boxplot of patient satisfaction with the outcome of each technique, evaluated using the Breast-Q^TM^ questionnaire

On average, patients reported 71 ± 27% satisfaction with the surgical outcome of treatment with lipograft. Patients who underwent implant augmentation were satisfied to the extent of 76 ± 19% (Fig. 5). Mann–Whitney testing revealed no significant difference in the two levels of satisfaction (*p* = 0.554).

**Fig. 6:**
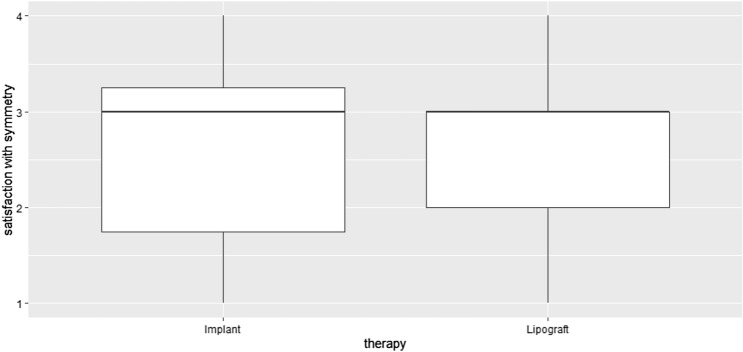
Boxplot of the patient satisfaction with breast symmetry, evaluated using a scale from 1 (least) to 4 (greatest)

The results of the additional question on satisfaction with breast symmetry (on a scale from 1 to 4) were 2.7 ± 1.1 for implant augmentation and 2.6 ± 1.0 for lipograft. The difference between satisfaction with the symmetry for both methods illustrated in Fig. 6 was not found to be significant (*p* = 0.69).

### Objective Outcome Evaluation

**Fig. 7:**
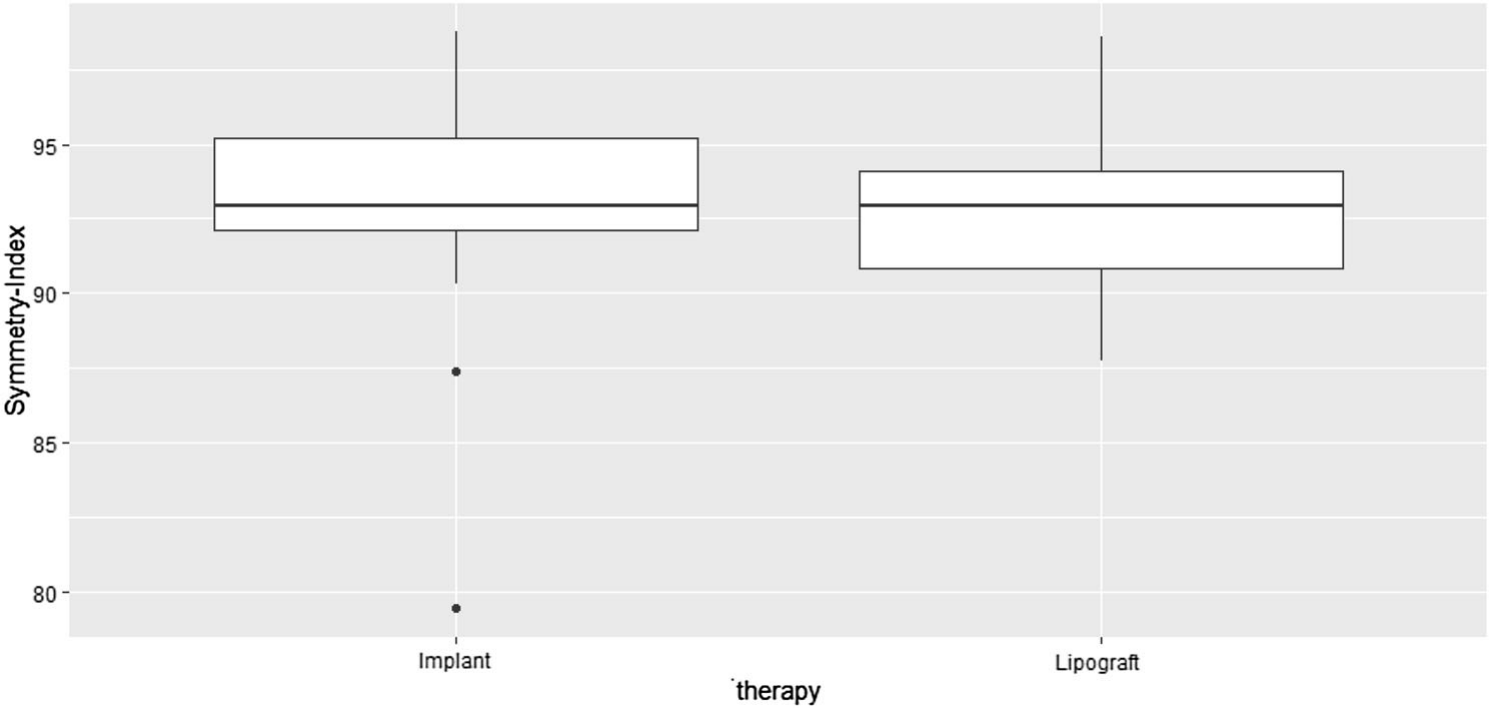
Boxplot comparing the SI calculated on patients treated with implant augmentation and lipograft

We evaluated the SI and the volume difference between both breasts postoperatively. As portrayed in Fig. 7, the SI was 93 ± 3 % for lipograft and 93 ± 5 % for implant augmentation. The difference between both methods was not statistically significant (*p*-value: 0.762).

**Fig. 8:**
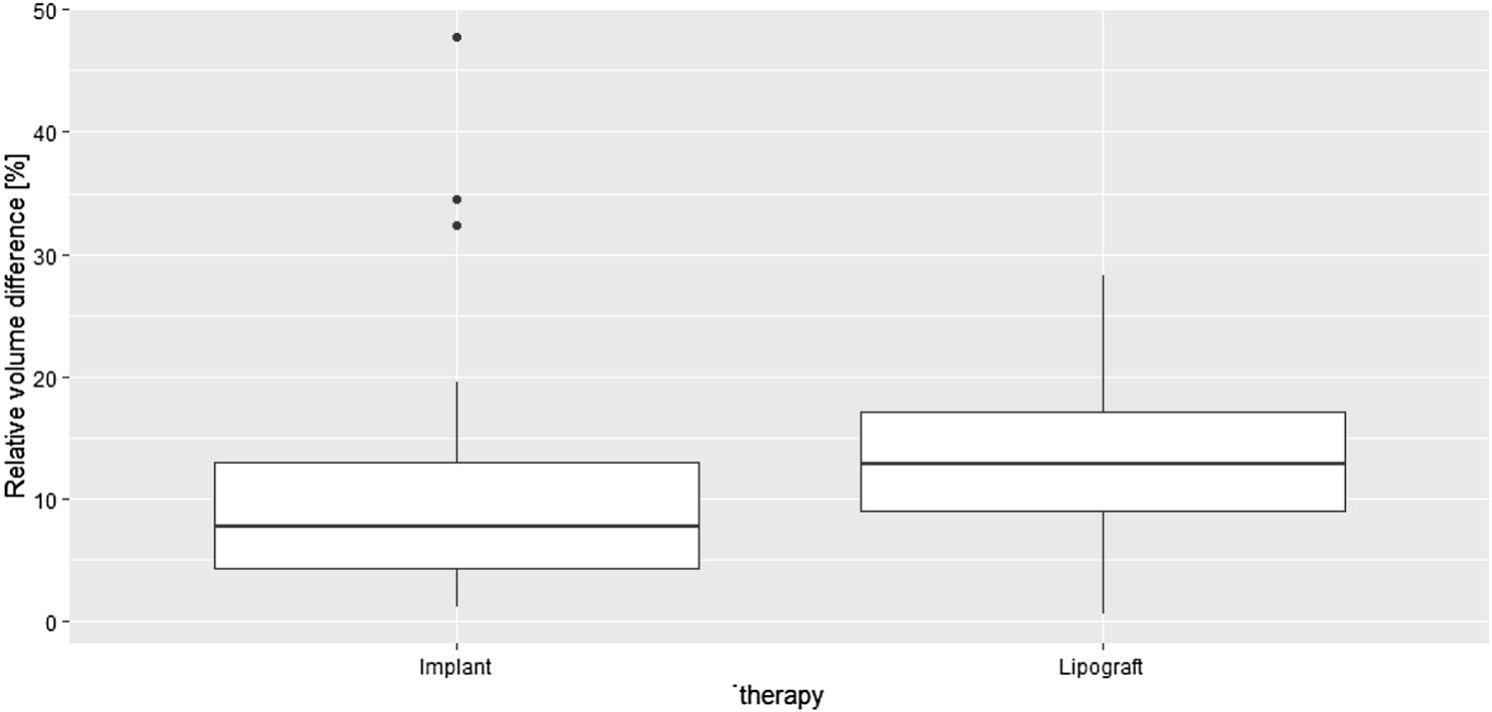
Comparison of the postoperative relative volume difference measured with the 3D-scanning system Vectra^®^

**Fig. 9:**
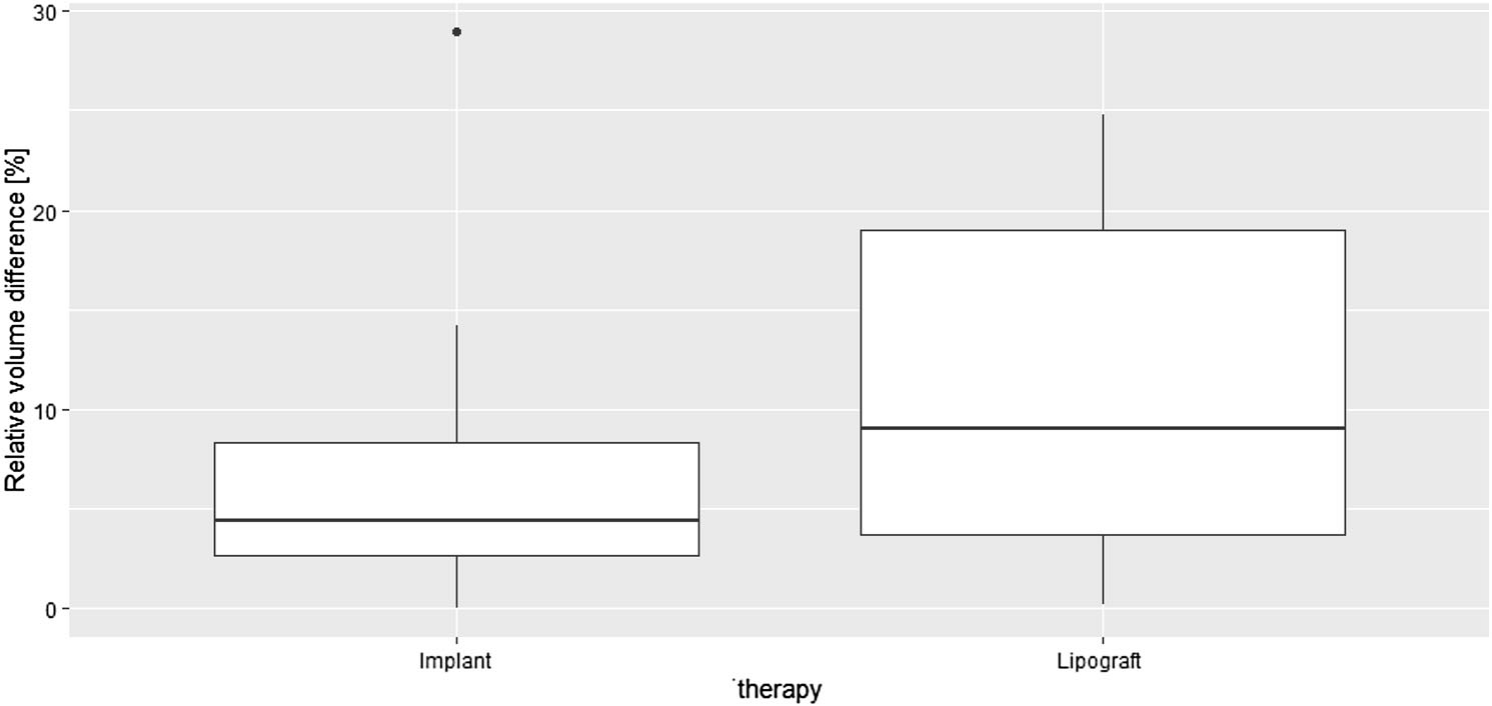
Comparison of the postoperative relative volume difference measured with the Breast-V formula

The Breast-V formula calculated a postoperative volume difference of 27 ± 30 g for implant augmentation and 50 ± 47 g for lipograft. In contrast, Vectra^®^ measured a postoperative volume difference of 74 ± 87 ml for implant augmentation and 86 ± 70 ml for lipograft. Neither the data collected with Vectra^®^ nor Breast-V were statistically significant (*p*-values 0.41 and 0.13, respectively). Small volume differences between breasts may be more noticeable in smaller than in larger breasts. The volume difference was therefore set in relation to the volume of the bigger breast of the patient. Figures 8 and 9 depict the results. It should be noted that the final volume achieved was similar in both the lipograft (424 ± 130 ml) and implant groups (390 ± 79 ml), which was measured using Breast-V.

## Discussion

In this study, we compared silicone implants and lipograft as techniques available to correct congenital breast asymmetry. Since surgery is usually performed early on in a patient’s life, long-term satisfaction with the results over many years is decisive. We thus evaluated the patients in our collective on average 6.8 years after their surgeries.

In order to measure the long-term patient-related outcome, we implemented the Breast-Q^TM^ questionnaire.

Lipograft and implant therapy yielded similarly good results (with an average subjective long-term outcome satisfaction of 74 % for the whole study population). We thus consider both therapies as current and relevant, confirming the findings of a study conducted by Sandsmark et al. [25], in which no significant difference between therapy with or without implants could be detected. Additionally, our study supports the results of Kuzbari et al. [26]. Although their study had a different focus and did not take lipograft into account, they also reported long-term satisfaction with the correction of congenital breast asymmetry, and general patient satisfaction with the long-term outcome.

A drawback of the Breast-Q^TM^ lies in the fact that the questionnaire lacks any question on satisfaction with breast symmetry after surgery. Hence, we added an additional question, which revealed equal results for both surgical methods. Although the data demonstrated large deviations, patients were satisfied on average with their breast symmetry in the long term. This is consistent with our objective results on symmetry, as our newly developed symmetry index revealed good results for both methods (93 %). The Department of Plastic Surgery at The University of Texas MD Anderson Cancer Center, Houston, found that a substantial proportion of women (50.6 % of a non-operated cohort) exhibit a volume difference of greater than 50 ml between the right and left breasts [27]. The MDACC study provides normative data on the extent of breast asymmetry in preoperative patients that can guide us in setting realistic goals for reconstruction procedures. Indeed, our postoperative cases demonstrate symmetries closely resembling those of natural breasts.

However, some patients reported postoperative numbness or hypersensitivity. This was also observed in other studies, including that by Heine et al. [28], who described less impairment of sensitivity of the breast after lipograft than after implant reconstruction. As previously described, the objective assessment of breast sensitivity is of great interest and should be evaluated in follow-up work [28].

Anthropometric measurements utilized in the Breast-V formula provide a valid approximation of the breast volume [9]. However, three-dimensional imaging with Vectra^®^ in combination with Breast-Sculptor^TM^ (Canfield Scientific, USA) is a validated method for calculating breast volumes as well. Indeed, we were able to reveal a good correlation of breast volume differences when comparing the Breast-V approximation and the three-dimensionally based calculation.

Of course, three-dimensional imaging is superior to anthropometric measurement, as it creates a digital twin, and thus allows for further comparisons without needing an additional, physical examination. This makes it possible to analyze even small volume deficits or scars, which are not represented by anthropomorphic measurements.

The oncogenic effects of fat grafting remain controversial [29, 30]. A retrospective study published in 2016 [31], which included 719 patients with benign and malignant breast disease and fat grafting, revealed no evidence of an increase in the incidence of locoregional recurrence, systemic recurrence, or new onset of breast carcinoma. Since breast augmentation with silicone implants presents long-term safety concerns (ALCL, capsular fibrosis, leakage) and forcibly leads to implant exchanges, it could prove advantageous to perform surgery on young patients with congenital breast asymmetry using lipograft. In addition, unilateral breast augmentation with silicone implants results in a difference in the feel of the breast and an asymmetry in the palpation findings.

Lipograft as means of breast augmentation required on average 2.9 sessions to achieve the desired result, which is significantly more than in the implant group (1.3 sessions). On average, both methods required a similar amount of operation time per session. As mentioned before, patients with congenital breast asymmetry are usually treated very early in their life, leading to several implant replacements. Hence, compared to lipograft, the number of operations required for implant augmentation is expected to be greater over the total lifetime of the patient. Furthermore, studies revealed that lipografts can be enriched with progenitor and stem cells using mechanical shear stress only, without causing any manipulation of the cells’ secretome [32]. This could improve the uptake of adipose cell transplantation and improve patient satisfaction with autologous fat transfer.

A limitation of this study is clearly its small sample size. Although the sample size is common for congenital breast asymmetry studies [26, 33, 34] and our two groups are very similar in terms of age and BMI, our collective size of 32 allows only limited generalizations. In the future, a multicenter approach may generate more focused results considering improvements in lipograft processing and lighter implants.

## Conclusions

In this study, we demonstrate that there were no significant differences in the satisfaction with long-term outcome between lipograft and silicone implant augmentation, either subjectively or objectively, in our study population. Both surgical procedures afforded good levels of satisfaction with surgical outcome and breast symmetry. Lipograft needed more sessions to achieve the desired result ab initio, but implant augmentation requires several implant replacements over the course of young patients’ lifetimes.

On the one hand, the tissue compatibility of breast implants may improve in the future and thus require fewer implant replacements. On the other hand, the uptake of adipose cell transplantation may improve as well, such that fewer surgeries will be necessary. Therefore, further prospective studies and investigations will be needed in the future to compare the competitive surgical techniques of breast augmentation in congenital malformations.
